# Evaluating the combined efficacy of Telisotuzumab Vedotin and artificial intelligence in the treatment of non-squamous non-small cell lung cancer: a narrative review focusing on pharmaceutical and technical insights

**DOI:** 10.3389/fonc.2025.1673586

**Published:** 2025-10-20

**Authors:** Syeda Huda Fatima, Syeda Jazilah Faisal, Syeda Verisha Batool, Mehak Faisal, Talha Aamir, Zain Ul Abideen Shahid, Raghabendra Kumar Mahato

**Affiliations:** ^1^ Department of Medicine, Dow University of Health Sciences, Karachi, Pakistan; ^2^ Department of Medicine, Gandaki Medical College Teaching Hospital and Research Center, Pokhara, Nepal

**Keywords:** Telisotuzumab Vedotin (Teliso-V), artificial intelligence (AI), non-squamous non-small cell lung cancer (NSCLC), c-MET overexpression, antibody-drug conjugate (ADC)

## Abstract

**Background:**

Non-squamous Non-small Cell Lung Cancer (NSCLC) is among the most common lung cancers that are therapy-resistant. Telisotuzumab Vedotin (Teliso-V), an antibody-drug conjugate (ADC), targets mesenchymal-epithelial transition factor (c-MET) high cells, with minimum side effects. Additionally, Artificial Intelligence (AI) aids in enhancing diagnosis, detection of mutations and advancing personalized care. Teliso-V, with the assistance of AI technologies such as radiomics, enhances efficacy against cancer.

**Objective:**

To assess the combined role of Teliso-V and AI in enhancing diagnosis, treatment, and outcomes in non-squamous NSCLC.

**Method:**

This review emphasizes the value of Teliso-V and the contribution of AI in enhancing the diagnosis and therapy of NSCLC. It is based on PubMed and ClinicalTrials.gov trials over the past two decades.

**Result:**

Teliso-V is effective in MET-high non-squamous NSCLC, yielding a response of 34.6% in the LUMINOSITY trial. Moreover, the combination with epidermal growth factor receptor inhibitors like Osimertinib and Erlotinib enhances outcomes, but the combination with immunotherapy (Nivolumab) provided negligible benefit. Moreover, AI has emerged as a powerful agent in cancer management, helping with diagnosis, foretelling mutations, and refining treatment regimens. It also maximizes Teliso-V use in NSCLC with improved patient selection, the ability to predict MET status from imaging and pathology, and the combination of circulating tumor DNA with radiomics for real-time tracking. Additionally, in silico experiments and machine learning algorithms optimize the sequence of treatment and reduce toxicity. Consequently, AI-driven Clinical Decision Support Systems in electronic medical records facilitate precision prescribing. Though challenges such as data bias and black-box decision-making occur, there is potential for AI to optimize personalized NSCLC therapy.

**Conclusion:**

Teliso-V is highly effective in MET-high NSCLC with tolerable side effects. Its combination with AI holds the hope of early diagnosis, individualized treatment, and intelligent ADCs of the future, but for this to manifest, clinical data and biomarker improvements must materialize.

## Introduction

Lung cancer is the most commonly diagnosed cancer, with GLOBOCAN 2022 reporting approximately 2.5 million new cases in 2022 (1). It also remains the leading cause of cancer-related mortality worldwide, resulting in an estimated 1.8 million deaths per year (2). Non-small cell lung cancer (NSCLC) is the most common malignancy associated with lung cancer, accounting for approximately 84% of all cases, of which the non-squamous variety remains the dominant type (3), and histological findings suggest that lung adenocarcinoma (LUAC) is the most pre-dominant subtype of NSCLC among the non-squamous variety (4). Most non-squamous NSCLC cases are usually diagnosed at an advanced stage and are typically associated with a poor prognosis (5). This demonstrates the fact that conventional treatment regimens such as chemotherapy, tyrosine kinase inhibitors (TKIs), and immunotherapies impose several limitations such as drug resistance, toxicity, and variable response rates, demanding the need for innovative therapeutic strategies along with technical insights to detect the disease in its early stages (6).

Over the years, NSCLC has undergone progressive pathological changes, developing complex molecular mechanisms that drive resistance to conventional therapies and underscore its marked heterogeneity, which remains a major challenge to effective diagnosis and treatment (7). Different patients may present with tumors driven by distinct genetic alterations such as EGFR, KRAS, or c-MET, each demanding a tailored therapeutic approach and complicating management with standard treatment strategies (8). Furthermore, recent studies highlight additional molecular drivers of resistance and heterogeneity in NSCLC. For example, the overexpression of the oncogene 5′–3′ exoribonuclease 2 (XRN2) has been shown to enhance EGFR signaling, promote epithelial mesenchymal transition (EMT), and facilitate metastatic spread, thereby contributing to more aggressive phenotypes and therapeutic resistance (9). Similarly, activation of the Wnt/β-catenin pathway has been linked to PD-L1 overexpression, which promotes immune evasion and resistance to EGFR-TKIs (10). Such findings reinforce the urgent need for advanced targeted therapies capable of overcoming these molecular escape mechanisms. Therefore, recent advancements in modern healthcare, such as targeted drug therapies, as well as the novel integration of artificial intelligence (AI) in the screening, early detection, and treatment of patients with non-squamous NSCLC, have been shown to have decreased the mortality trends among the patients by 6.3% annually from 2013 through 2016 (11). Additionally, the long-term survival outcomes of these patients have significantly improved from 8% in 2008 to 25% in 2020 (7).

Telisotuzumab Vedotin (Teliso-V), an anti-MET antibody-drug conjugate (ADC) targeting EGFR-mutant, c-MET-overexpressing non-squamous NSCLC, is proving to be one of the most promising contributors to the declining mortality rates of these cases (12). It comprises the monoclonal antibody telisotuzumab, conjugated to the microtubule inhibitor monomethyl auristatin E (MMAE) via a cleavable dipeptide linker (13). The c-MET protein, a transmembrane tyrosine kinase receptor activated by its ligand, hepatocyte growth factor (HGF), is essential for regulating various cellular processes (14). Its overexpression has been linked to approximately 50% of non-squamous NSCLC cases (15). Teliso-V targets tumor cells overexpressing c-MET by binding with them and releasing cytotoxic components designed specifically to target them, thereby minimizing off-target toxicity as would be in the case of conventional chemotherapy (16). Current studies highlight the promising efficacy of Teliso-V and its future benefits in the treatment of non-squamous NSCLC, emphasizing the key advancements made in the field of precision oncology (17).

The integration of AI in the field of precision medicine has revolutionized the way healthcare professionals approach lung cancer, and it is proving to be a key driver in the increased efficacy of patients suffering from non-squamous NSCLC (6), as it plays a crucial role in cancer diagnosis as well as imaging, efficacy evaluation, survival prediction, early detection and histopathological findings (18). Moreover, AI models can efficiently process substantial amounts of data, providing clinicians with improved diagnostic accuracy to treat cancer patients via a combination of different algorithms such as machine learning (ML) and deep learning (DL) models for mutation prediction and automated analysis of complex imaging and genomic datasets (19), radiomics for quantitative assessment of tumor heterogeneity from radiological scans (20), multi-omics integration for combining genomic, proteomic, and imaging data (21), natural language processing (NLP) for extracting insights from clinical narratives (22), and in silico trials for simulating patient-specific treatment outcomes (23). By using specific AI-driven tools like trained neural networks and support vector machine classifiers, AI enables efficient analysis of radiological and molecular profiles of patients having unique biomarkers and genetic mutations, including EGFR-mutant and c-MET overexpressing genes (24, 25). AI assists in targeting NSCLC patients by non-invasively predicting c-MET overexpression status; DL models applied to hematoxylin and eosin (H&E) stained histopathology slides help in the classification and mutation prediction of NSCLC (26) as well as radiomics enables the extraction of high dimensional quantitative image features from routine imaging such as CT or PET scan that can capture tumor heterogeneity, enabling precise patient selection and stratification for targeted drug therapies while reducing the need for invasive biopsies (27). Thus, these technical insights can be useful in the treatment of non-squamous NSCLC by complementing them with modern treatment options like Teliso-V, through identifying the disease early in its stage and providing suitable personalized dosing regimens to minimize limitations.

In order to tackle the challenges and obstacles imposed by non-squamous NSCLC, there is an urgent need to explore new, advanced, targeted, and personalized treatment strategies. Thus, by incorporating the use of AI in the management of these cases, optimal therapeutic effects may be achieved from targeted drug therapies like Teliso-V. This narrative review aims to provide a comprehensive overview of the pharmaceutical insights provided by Teliso-V along with the technical advancements made by AI in the management of non-squamous NSCLC and explore how these innovations are leading us to a new era of precision medicine.

## Methodology

In this narrative review, we aimed to explore the management of NSCLC by using Teliso-V and AI. We examined the pharmacologic profile and clinical outcomes of Teliso-V. We also found out how AI can improve diagnosis. In addition, it also discusses how AI can help in patient selection and personalized treatment strategies. Further evaluated whether it improved therapeutic efficacy or not. This review examined articles from PubMed and Google Scholar over the past two decades. Moreover, additional data were retrieved from clinical trial websites (e.g., ClinicalTrials.gov) and from regulatory authorities. This includes the U.S. Food and Drug Administration (FDA) and the European Medicines Agency (EMA), include the latest available information on Teliso-V and AI. Searching included a combination of relevant words and MeSH terms. These include “Telisotuzumab Vedotin (Teliso-V)”, “non-squamous NSCLC”, “c-MET overexpression”, “antibody-drug conjugate (ADC)”, “machine Learning” and “in silico trials”. Inclusion criteria comprised randomized controlled trials, systematic reviews, meta-analyses, observational studies, clinical guidelines, and high-quality narrative reviews. Eligible studies were required to be published in peer-reviewed journals and written in English. These studies should report on Teliso-V or AI applications in NSCLC. Exclusion criteria included studies on squamous cell carcinoma and pediatric patients. Furthermore, non-human models, or conference abstracts without full data were excluded too. Titles and abstracts were screened for relevance. Full texts of potentially eligible articles were reviewed to ensure they met the inclusion criteria. 107 studies met the eligibility criteria and were included. Articles were chosen for their importance and science. Studies were also selected if they add value to the understanding of clinical, microbiological, and pharmacological characteristics of Teliso-V. Additionally, studies demonstrating the potential of AI-enhanced application of Telisotuzumab Vedotin (Teliso-V) in the targeted management of NSCLC were selected.

## Telisotuzumab Vedotin: mechanism and clinical development

Telisotuzumab Vedotin (Teliso-V), previously known as ABBV-399, is a novel ADC developed for the treatment of NSCLC (28). It is composed of the humanized monoclonal antibody ABT-700 conjugated to the monomethyl auristatin E (MMAE), a cytotoxic agent, through a cleavable valine–citrulline linker, forming an ABT-700–vcMMAE (28, 29).

The mesenchymal-epithelial transition factor (c-MET) is a receptor tyrosine kinase found on epithelial cells. It plays an important role in regulating wound healing and tissue remodeling processes under normal physiological conditions (30, 31). When HGF binds to the c-MET receptor, it induces dimerization and autophosphorylation of the receptor at tyrosine residues. Additionally, it creates docking sites for various signaling molecules like GRB2, PI3K, and STAT3 and triggers various pathways for the growth, proliferation, and survival of cells (31). MET, a proto-oncogene, encodes c-MET, which is composed of α and β chains linked by disulfide bonds. The β chain contains the signaling domain. HGF is activated by proteolysis and initiates the downstream signaling pathways. MET exon 14 encodes a regulatory juxta membrane domain that limits MET signaling. The mutation and deletion of this gene prevent proper receptor degradation, causing sustained MET activation and promoting uncontrollable cell proliferation and tumor growth (32, 33).

Overexpression and dysregulation of c-MET are frequently observed in NSCLC and are considered important therapeutic targets. In a study of NSCLC cases, 17% of tumors showed MET overexpression and 2.4% of patients had MET amplification. Overexpression was common in adenocarcinomas and female patients (34). Another study showed that NSCLC patients expressed HGF-α (67.3%), c-Met (74.3%), and VEGF-C (65.5%), significantly higher than the normal lung tissue levels (20.4%, 23.0%, and 23.9%, respectively) (35). Another study showed 95% of NSCLC patients had varying levels of c-MET expression, with 27% (+), 36% (++), and 32% (+++). Among genetically tested patients, epidermal growth factor receptor (EGFR) mutations were the most common (49%), followed by wild-type (37%) (36).

As described above, Teliso-V binds with high affinity to tumor cells expressing c-MET. Upon binding, the ADC is internalized by the tumor cells and delivers the MMAE directly into the cell. MMAE is released intracellularly by the proteolytic cleavage of the valine–citrulline linker. It then binds to tubulin protein, disrupting mitosis and causing the tumor cells’ death (28, 29). ABT-700 has shown limited efficacy in tumors that overexpress c-MET without gene amplification, whereas it’s combination with a valine–citrulline linker and MMAE in Teliso-V has demonstrated significantly higher efficacy. However, Teliso-V remains effective in c-MET expressing tumors regardless of MET gene amplification status. ABT-700 has been evaluated by enzyme-linked immunosorbent assay (ELISA) and fluorescence-activated cell sorting (FACS) to examine its binding characteristics. ABT-700 binds to c-MET with an affinity of 0.22 nanomolar and shows comparable binding to c-MET from cynomolgus monkeys, but not to mouse-derived c-MET. Furthermore, both hepatocyte growth factor (HGF)-dependent and HGF-independent c-MET signaling are blocked by ABT-700 (28).

A study showed that XRN2 overexpression increased cluster of differentiation 31 (CD31) levels in lung metastatic lesions. Additionally, XNR2 promoted migration, invasion, and epithelial–mesenchymal transition (EMT). This triggered epidermal growth factor receptor (EGFR) phosphorylation, activating its downstream signaling pathway (9). In NSCLC cells, normal (wild-type) EGFR can also trigger the phosphorylation and activation of c-MET (37). Hence, teliso-V by blocking c-MET signaling, may reduce the downstream effects of XRN2. This makes the tumor less able to invade, metastasize, or form new blood vessels. Autophosphorylation of c-MET can activate the Extracellular signal-regulated kinase (ERK) (38). ERK, in turn, phosphorylates Oncoprotein 18 (Op18)/stathmin, a protein that controls microtubule stability through phosphorylation, affecting cell behavior and survival. Teliso-V blocks c-MET, indirectly reducing (Op18)/stathmin activity and causing cell death (39). Therefore, teliso-v may help overcome Taxol resistance, a drug that exerts its cytotoxic effects partly by targeting Op18/stathmin and inducing apoptosis.

**Figure 1:**
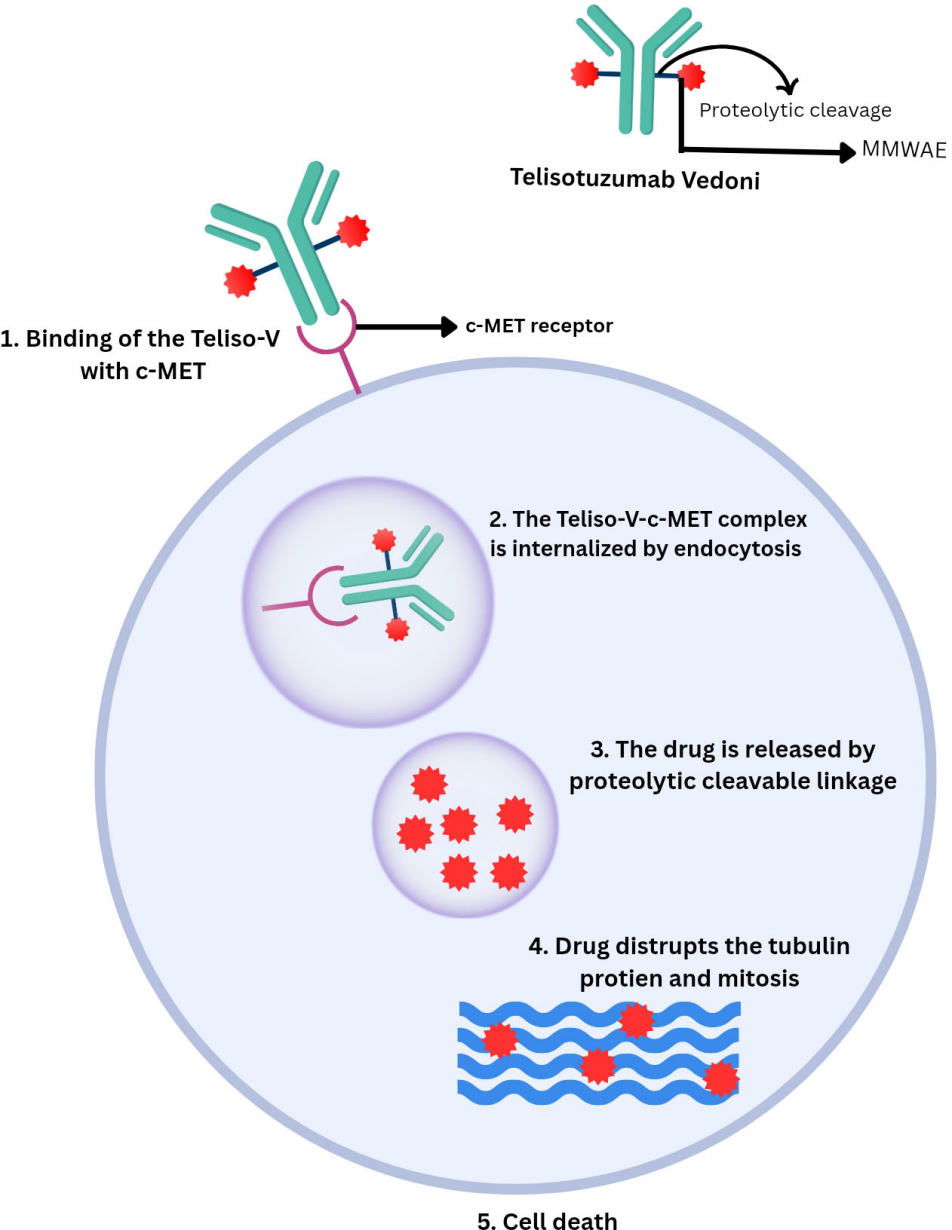
Mechanism of action of Telisotuzumab Vedotin.

Several trials and studies have been conducted to evaluate the use of Teliso-V in the treatment of NSCLC. A phase I study showed that Teliso-V at 2.7 mg/kg was tolerable with manageable toxicities. Some efficacy was observed in c-Met–positive NSCLC (13). Another phase I trial showed Teliso-V was well tolerated. At recommended doses (≥1.6 mg/kg Teliso-V once every 2 weeks), it showed a 23% response rate with durable responses (16). Moreover, the Phase II LUMINOSITY trial evaluated Teliso-V in patients with NSCLC with c-MET overexpression and showed that Teliso-V produced durable responses in patients, particularly in those with high c-MET expression (40). Additionally, three phase Ib trials also showed the effectiveness of the combination of Teliso-V with erlotinib, osimertinib and nivolumab in the treatment of NSCLC (41–43). All of these combinations are well-tolerated. As shown in **Figure 1**.

## Artificial intelligence in NSCLC treatment optimization

Artificial intelligence (AI), more specifically through machine learning (ML) and natural language processing (NLP), is transforming the care of NSCLC by greatly improving the accuracy of histological subtype classification, molecular profiling, and treatment personalization (44). Integration of AI into radiomics, genomics, and pathology interpretation is transforming traditional workflows to provide more precise, efficient, and personalized care. Radiomics transforms imaging information—e.g., CT or PET/CT scans—into high-dimensional, quantifiable features with automated algorithms. Such models can non-invasively extract subtle intra- and peri-tumoral features, providing reproducible and cost-efficient insights into lung cancer and its tumor microenvironment (45). Radiomics-enabled ML or DL models are currently well established in every phase of lung cancer management—diagnosis, staging, treatment planning, and prognosis—with performance equal to or even superior to that of radiologists (46). In the field of genomics, AI facilitates early prediction of driver mutations like EGFR and KRAS. EGFR mutations are associated with improved response to erlotinib and gefitinib, while KRAS mutations indicate therapeutic resistance. Early detection of these mutations using AI-based, image-guided prediction allows targeted therapy and enhanced outcomes. While conventional methods are effective, they tend to be invasive and time-consuming. In contrast, AI-based image-guided mutation prediction offers a promising, less invasive alternative (20). In pathology, AI aids in the evaluation of biopsy and resection slides to subtypes such as LUAD that require unique treatment strategies. According to studies, image features learned through AI can be used to predict prognosis and support personalized care (47). AI is also applied to non-invasive diagnostics, such as electronic noses (e-noses) that detect NSCLC from volatile organic compounds in breath, with advanced models like SIR-3DCNN optimizing sensor arrays and using 3D convolutional networks, achieving 100% sensitivity and 92.94% accuracy, demonstrating potential for early, real-time lung cancer detection (48)​. Therefore, AI greatly improves diagnostic accuracy, clinical workflows, and precision oncology in NSCLC.

Immunotherapy has revolutionized the management of NSCLC, but not all patients are equally benefited (49). Pooled analyses of randomized trials show that neoadjuvant or perioperative immunotherapy improves pathological response rates and clinical outcomes in resectable NSCLC. Subgroup analyses suggest benefits for different patient populations, which supports its potential as a standard perioperative strategy​ (50)​. Predictive biomarkers are necessary to determine responders. Immune checkpoint inhibitors (ICIs) against PD-1/PD-L1 have emerged with striking survival advantages in clinical trials, with PD-L1 as the prime biomarker. The evaluation of PD-L1 by immunohistochemistry (IHC) is plagued by interobserver variability, influencing treatment choice. As a solution, AI and digital pathology provide standardized tools for precise PD-L1 assessment (51). Likewise, tumor mutational burden (TMB), another immunotherapy biomarker, cannot be effectively assessed by whole-exome sequencing because of its cost and complexity. To address this challenge, AI-driven models such as Image2TMB can predict TMB from standard histopathology images (52). In addition, defining the tumor microenvironment (TME), a factor that plays an essential role in tumor progression and response to treatment, is hampered by conventional approaches. In this regard, AI combines disparate data sources, such as imaging and omics, to evaluate the TME more effectively. Moreover, DL enables AI to chart immune cell distribution, reveal biological patterns, and facilitate personalized treatment planning (53).

In silico trials, also known as virtual trials, are computer-simulated clinical studies designed to evaluate the safety and effectiveness of treatments through computer simulation and modeling. They have been stimulated by the rapid growth in AI and precise computational models (23). The In-silico trials synthesize patient-specific and disease-specific data to generate individualized virtual cohorts by drawing on real-world clinical-genomic data. These systems mimic human pathology and physiology, such as genetic differences influencing clinical responses. In this aspect, AI allows for the merging of 3D anatomical shapes, biochemical pathways, and gene networks. In addition, Causal AI methods mimic control and efficacy arms, direct patient recruitment, titrate regimens to optimize, and facilitate precision subgroup analysis (54). While targeted therapies have enhanced survival in patients with NSCLC and actionable biomarkers, the relative 5-year survival rate for all patients is less than 20%. Historically, biomarkers have been found utilizing linear statistical methods and are presumed to be mutually exclusive in clinical decision-making. Recent data indicate that more than one actionable biomarker can exist within a single tumor and thus requiring models that are able to recognize linear and non-linear patterns. AI/ML algorithms provide this flexibility and have promise in biomarker discovery from high-dimensional data. A few AI-powered devices already have Food and Drug Administration’s (FDA) approval for performing activities such as tissue segmentation by machine and feature extraction from lung CT scans. Deep learning (DL) techniques also advance whole-slide image analysis in digital pathology (55). AI pre-screening with digitized H&E slides can potentially enhance MET detection and decrease dependence on precious IHC and tissue samples. For example, a convolutional neural network (CNN)-based deep learning model that accurately predicted MET overexpression in lung adenocarcinoma from routine histopathology slides (56). Similarly, a logistic regression model using radiomics-style morphological features (cell shape, texture, grayscale intensity) extracted from whole-slide images, achieving an AUC of 0.77 for identifying MET amplification and exon 14 skipping alterations (57). **Table 1** below summarizes AI applications in NSCLC showing diagnosis, biomarker prediction and treatment optimization.

**Table 1:** Summary of AI applications in NSCLC.

Domain	AI application	Examples/Models	Advantages
Diagnosis	Radiomics (CT, PET/CT)	Radiomics-enabled ML/DL models	Non-invasive, reproducible, cost-efficient; improves diagnosis, staging, treatment planning, prognosis; equal/superior to radiologists
	Pathology	AI evaluation of biopsy/resection slides; LUAD classification; AI-learned image features predicting prognosis	Improves diagnostic accuracy, supports personalized care
	Non-invasive diagnostics	Electronic noses (e-noses), SIR-3DCNN, 3D CNN	Optimized sensor arrays (22→8); converts multivariate time series (MTS) to MTMIs; achieves 100% sensitivity, 92.94% accuracy; enables early, real-time NSCLC detection
	FDA-approved tools	AI-powered devices approved for tissue segmentation and CT feature extraction	Streamlines workflows
Biomarker Prediction	Genomics	AI predicting EGFR and KRAS mutations from imaging	Early, less invasive, faster than conventional methods; enables targeted therapy and better outcomes
	Immunotherapy biomarkers	- PD-L1: AI/digital pathology for standardized assessment - TMB: *Image2TMB* predicting TMB from histopathology	Reduces interobserver variability in PD-L1 IHC; avoids costly/complex WES for TMB
	Predictive biomarkers for therapy response	Pooled analyses of randomized trials with AI integration for neoadjuvant or perioperative immunotherapy	Determines responders; improves pathological response rates and clinical outcomes; supports standard perioperative strategy; benefits different patient subgroups
	Tumor Microenvironment (TME)	AI integrating imaging + omics; DL for immune cell distribution	Defines TME more effectively; reveals biological patterns; aids personalized treatment planning
	Biomarker Discovery	AI/ML detecting >1 actionable biomarker within single tumor	Recognizes linear & non-linear patterns; flexible vs linear statistics
Treatment Optimization	In-silico Trials	AI-driven virtual trials; causal AI methods for patient recruitment, regimen titration, subgroup analysis	Individualized virtual cohorts; mimics human pathology/physiology; facilitates precision medicine
	Pre-screening	AI using digitized H&E slides for MET detection	Enhances MET detection; decreases dependence on IHC/tissue samples
	Digital Pathology	DL advancing whole-slide image analysis	Enables precise feature extraction

ML software can contain biases that amplify stereotypes and healthcare inequities based on imperfections in electronic health records that are tied to race, gender, age, insurance, and socioeconomic status. Those biases impact big groups of people around the world, with profound social and economic implications (58). One of the biggest worries is the “black box” model of medical AI—its decision-making process is inscrutable, so patients, doctors, and even the developers of the software often have no understanding of how treatment recommendations are generated. This unexplainability will potentially do more harm than human error, as it undermines patient autonomy to participate in shared decision-making and causes tremendous psychological and financial tolls on patients (59, 60). AI technologies, although in their nascent stage, are gaining much attention in oncology for their capability to personalize the management of NSCLC, as they address the complexity of the disease and facilitate data-driven, informed decision-making. Challenges remain despite such efforts, but validation across institutions and populations is crucial to facilitate real-world clinical adoption (45).

## Clinical efficacy of Teliso-V and AI

As mentioned above, c-MET protein, a transmembrane receptor tyrosine kinase, is overexpressed in approximately 50% of NSCLC patients. Teliso-V is an ADC that targets c-MET to treat NSCLC (12). Its efficacy is shown in MET-high NSCLC patients in phase I and II trials, particularly in non-squamous EGFR- wildtype subgroup, as summarized in **Table 2**.

**Table 2:** Summary of key clinical trials of Telisotuzumab Vedotin in NSCLC.

Trial name	Phase	Patient type	ORR (%)	PFS (months)	OS (months)	References
LUMINOSITY	II	c-MET+, non-squamous EGFR-wildtype NSCLC	28.6 (overall); 34.6 (c-MET high); 22.9 (c-MET intermediate)	5.7 (overall); 5.5 (c-MET high); 6.0 (c-MET intermediate)	14.5	(40)
First-in-Human Dose Escalation Study	I	Advanced NSCLC	–	–	–	(13)
Study of 2 or 3-week Dosing	I	c-MET+ NSCLC	22	5.2	–	(16)
Teliso-V + Osimertinib	Ib	c-MET+, EGFR mutant advanced NSCLC	50	7.4	–	(42)
Teliso-V + Erlotinib	Ib	EGFR mutant, c-MET+ NSCLC	30.6 (Total); 32.1 (EGFR+); 62.5 (MET-advanced)	5.9	–	(41)
Teliso-V + Nivolumab	Ib	Advanced NSCLC	7.4	7.2	–	(43)

The key trial that evaluated the efficacy of Teliso-V is the phase II LUMINOSITY trial, where 172 patients with non-squamous EGFR-wildtype were treated with Teliso-V in 2 stages, with a median follow-up of 20.2 months in c-MET high patients. In stage I, patients with c-MET overexpressing NSCLC were identified, whereas in stage II the efficacy was evaluated by administering Teliso-V at 1.9 mg/kg once every 2 weeks (q2w). The primary endpoint, objective response rate (ORR) was 28.6% overall, with 34.6% for c-MET high and 22.9% for c-MET intermediate. Meanwhile, the secondary endpoints included progression-free survival (PFS) of 5.7 months overall, with 5.5 and 6.0 months for c-MET high and intermediate, respectively. The median overall survival (OS) of 14.5 months and the duration of response (DOR) of 8.3 months (40).

Prior to that, a phase I First-in-Human Dose-Escalation trial assessed the dosage and scheduling. Teliso-V was given as monotherapy intravenously at 3.0 mg/kg and 3.3 mg/kg once every 3 weeks (q3w) to 9 and 3 patients, respectively, with one in each category experiencing dose-limiting toxicity. However, it was well-tolerated at 2.7 mg/kg q3w and hence it was the recommended phase II dose (RP2D) (13). Another phase I trial evaluated Teliso-V monotherapy given either q2w (1.6-2.2 mg/kg) or q3w (0.15-3.3 mg/kg), in 28 and 24 patients, respectively, out of a total of 52 NSCLC patients. Of these, 40 were c-MET+ and included in the efficacy-evaluable population. On the basis of safety and antitumor activity in c-MET+ NSCLC shown, RP2D was established at 1.9 mg/kg q2w and 2.7 mg/kg q3w. The median PFS was 5.2 months and ORR in c-MET H-score ≥ 150 patients was 22%, underscoring the importance for MET stratification (16).

Combination strategies were established and evaluated as part of the phase Ib trials. When given in combination with osimertinib (80 mg OD), Teliso-V (1.6 mg/kg or 1.9 mg/kg), resulted in an ORR of 50% and median PFS of 7.4 months, in 38 patients, even showing effectiveness in osimertinib-refractory cases (42). Another study with erlotinib (150 mg OD) in 42 EFGR-mutation NSCLC patients demonstrated an ORR of 30.6% overall; 32.1% in EGFR+ and 62.5% in MET-advanced subgroup, along with a median PFS of 5.9 months and disease-control rates (DCR) of 86.1% in EGFR+ and 100% in MET-advanced subgroup (41). However, phase Ib trial combining Teliso-V (1.6, 1.9 or 2.2 mg/kg q2w) with nivolumab (3 mg/kg, 240 mg), an ICI, did not show a promising result with an ORR of 7.4% and median PFS of 7.2 months in 27 patients. This shows the combined strategy did not improve outcomes as the pharmacokinetic profile was similar to Teliso-V monotherapy, despite the theory that Teliso-V-induced tumor death should improve antigen release and work well with PD-1 inhibition. The lack of PD-L1-based patient selection and low baseline T-cell infiltration in MET+ malignancies may be the cause of the limited effect. The overall benefits were less and the side effects remained (43). When assessing potential immunotherapy combinations, our results highlight the necessity of biomarker-driven methodologies and logical trial design, such as integrating immune-infiltration profiling or dynamic biomarkers.

Across multiple trials, the most common adverse events (AE) include peripheral sensory neuropathy, nausea, decreased appetite, vomiting, peripheral edema and fatigue (13, 16, 40). Grade ≥ 3 AEs were less frequent but included peripheral neuropathy (7% in LUMINOSITY), anemia, fatigue, hypoalbuminemia and fatigue, (4% each in phase I) and pulmonary embolism (8% in combination with osimertinib and 14% with erlotinib) (13, 40–42). In comparison of dosing schedules, q2w dosing regimens had higher rates of AEs including neuropathy and resulted in dose-reduction in 25% and interruption in 54% patients as compared to q3w regimen which resulted in dose-reduction in 13% and interruption in 38%, respectively (16).

Treatment discontinuations were not uncommon with 21.5% in LUMINOSITY trial due to AEs like peripheral sensory neuropathy (7%) and pneumonitis (7.6%) (40). Moreover, discontinuations were also reported in nivolumab combinations due to dose-limiting toxicity and in combination with erlotinib due to neuropathy (26%) (41, 43). This outlines the need for optimum dosing regimens to minimize the toxic findings.

While most of the trials gave evidence for Teliso-V’s clinical benefit, LUNG-MAP S1400K study, which enrolled patients with c-MET+ squamous NSCLC, reported that DCR was 52% and the PFS and OS were 2.4 and 5.6 months respectively, showing limited efficacy in the squamous subtype. Thus, the study supports the effectiveness of Teliso-V in the non-squamous subtype but not the squamous (61).

Artificial Intelligence (AI) is a newly emerging tool in treatment of NSCLC; from therapy selection to prediction of responses, AI has multidimensional benefits. DL models have shown potential in predicting response to ICI in advanced NSCLC patients using H&E stained slides. Area under curve (AUC) for ORR prediction were 0.75 in internal test and 0.66 in validation cohort. Moreover, DL models have proven to be beneficial over TMB, tumor-infiltrating lymphocytes (TILs) and PD-L1, and when combining with PD-L1, the AUC upgraded to 0.70 and the response rate to 51%, supporting multi model approach (62).

Circulating tumor DNA (ctDNA) assessed via mPCR-based assay has shown a strong association between detection and relapse risk in early-stage NSCLC, and hence shows a potential for early risk stratification and real-time monitoring (63). Another trial integrated blood-based TMB (bTMB) with DL model and ctDNA clearance showing improved AUC of 0.820 as compared to 0.703 (DL model alone) in predicting response to neoadjuvant immunotherapy (64).

TMB radiomic biomarkers (TMBRB) have shown potential in predicting TMB and response to immunotherapy, with AUC of 0.85 (training) and 0.81 (validation). Moreover, combining with Eastern Cooperative Oncology Group (ECOG) improves performance status and shows a better prediction of OS (p=0.007) and PFS (p=0.003) (65). Additionally, combination of radiomics (Rad), DL and Clinical with an AUC of 0.88 is an emerging non-invasive approach for presurgical decision-making in NSCLC (66).

AI models have also demonstrated capability in defining dosing schedules and predicting toxicity in NSCLC patients using machine learning (ML) (67). Another model derived from ML, mass-spectrometry based proteomic signatures, demonstrated improved PFS and OS in NSCLC patients treated via immunotherapy, proving it has the potential for better survival outcome (68).

There are certain drawbacks that affect the implementation of Teliso-V and AI despite their numerous benefits. Most Teliso-V trials have a limited sample size, exploratory nature, and exclude the comparator arms (13, 40). Moreover, c-MET+ had a predefined H-score of ≥ 150, limiting the patient number (16). Additionally, achieving AUC score of above 0.8 was difficult in all DL models, indicating the need of refinements to enhance accuracy. A major challenge faced by AI models in oncology is validation when the performance declines in external cohorts as compared to internal training sets (62). In addition, many models are trained on narrowly defined or single-institution datasets, limiting dataset diversity and reducing applicability across different patient populations (64). This highlights the concerns regarding generalizability and widespread adoption of these models. Furthermore, TMBRB was assessed in patients with early-stage NSCLC; further studies are needed to identify TMB levels among patients with advanced NSCLC and to explore the clinical utilization of ctDNA to advise management (65).

## Combining Teliso-V and AI for improved outcomes

Artificial intelligence (AI) brings an innovative approach in patient selection for Teliso-V, especially effective in NSCLC patients with MET overexpression or MET exon 14 skipping mutations. In a Phase I study, Teliso-V illustrated an ORR of 23% in MET-positive NSCLC patients, with a median DOR of 8.7 months, highlighting the need for accurate patient classification (16). Conventional diagnostics like IHC and PCR are usually limited due to tumor heterogeneity and observer bias (69). In this regard, AI, particularly ML and DL, enhances selection by integrating multi-omics data, i.e., genomics, transcriptomics, proteomics, imaging, and clinical records into predictive models (70). Most of these investigations have been retrospective in design, usually using datasets such as The Cancer Genome Atlas Program (TCGA) (71). Besides that, Convolutional Neural Networks (CNNs) have been used in pathology slides for mutation prediction, while ensemble methods like random forest (RF) and gradient tree boosting (XGB) are commonly used in radiomics for response classification (26, 72). Additionally, reinforcement learning methods, particularly deep Q-learning, have been investigated *in silico* for adaptive therapy sequencing. For instance, in NSCLC, a quantum deep reinforcement learning framework that uses deep Q-networks in a virtual radiotherapy setting has been shown to improve dose adaptation decisions, improving local control and reducing treatment toxicity (73).

Studies reveal that DL models help in predicting MET RNA overexpression directly from H&E-stained pathology slides with high accuracy, providing non-destructive, scalable screening tools (56). Furthermore, modern DL frameworks such as the SIR-3DCNN model, which applies 3D convolutional neural networks to multivariate time-series imaging data, show the potential for dynamic AI-based classification in the detection of lung cancer (48). Similarly, ML-driven radiomics improves selection by identifying subtle imaging and clinical features associated with treatment response (74). Combination of multi-omics data often depends on fusion strategies like autoencoders or late-fusion neural networks, to harmonize genomic, transcriptomic, and proteomic inputs into joint embeddings (75). Moreover, radiomic features including tumor heterogeneity, edge sharpness, and entropy are extracted from CT or PET scans and classified using algorithms such as SVMs or XGBoost (76). Additionally, many of these approaches have been validated externally on independent NSCLC cohorts, including TCGA and international radiogenomic repositories, revealing that the results are consistent and applicable in different contexts (77). These AI-enabled approaches reduce empirical treatment decisions, maximizing therapeutic benefit and advancing the paradigm of precision oncology.

**Figure 2:**
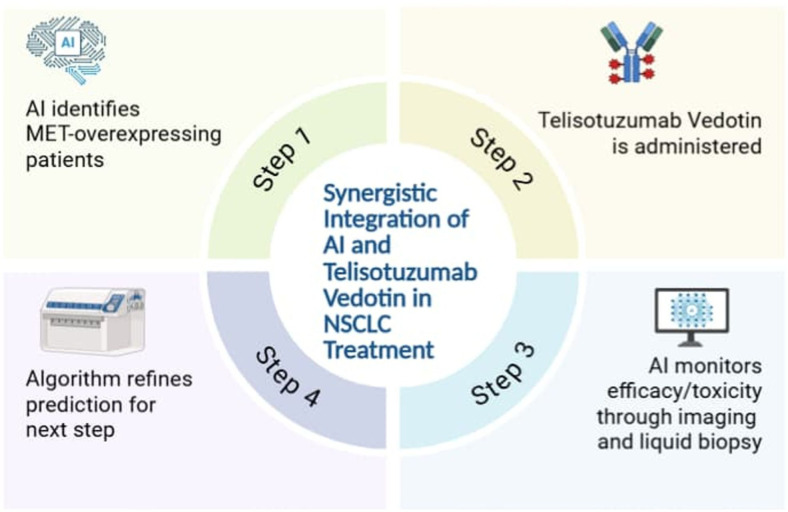
The synergistic integration of AI and Teliso-V in NSCLC treatment.

Additionally, the combined use of AI and Teliso-V in NSCLC treatment has been demonstrated below in **Figure 2**.

In NSCLC, the conventional Response Evaluation Criteria in Solid Tumors (RECIST) is usually unable to detect early or atypical responses to ADCs like Teliso-V, especially in case of necrosis or delayed tumor shrinkage (78). Combining ctDNA analysis with AI-supported imaging provides a strong, non-invasive approach. Studies suggest that serial ctDNA profiling using next-generation sequencing (NGS) in MET-positive NSCLC patients who are being treated with Teliso-V reveals real-time mutational changes and aligns with treatment response, promoting its use in tracking tumor evolution (79) Besides this, a meta-analysis evaluating over 3,000 advanced NSCLC patients confirmed that ctDNA clearance during therapy strongly predicted improved PFS and OS (80). Additionally, radiomics enables the extraction of quantitative features, such as tumor texture and heterogeneity, from CT or PET-CT scans, and AI algorithms can detect early imaging changes before anatomical size reductions occur. Moreover, a study of NSCLC patients undergoing chemoradiotherapy further revealed that when combined, ctDNA and radiomics improve the accuracy of prediction (81).

Furthermore, Teliso-V has shown potential in NSCLC treatment when combined with other modalities such as immune checkpoint inhibitors, i.e., anti-PD-1, chemotherapy, or EGFR-TKIs (43). Preclinical models and early-phase trials reveal that Teliso-V may synergize with immune therapies by increasing tumor immunogenicity through ADC-induced cell death and antigen release, potentially priming tumors for immune activation (43). Nevertheless, adverse events remain a concern. In Teliso-V monotherapy, the most frequent grade ≥3 toxicities include peripheral neuropathy, neutropenia, and pulmonary adverse events such as interstitial lung disease (40). When combined with checkpoint inhibitors such as nivolumab, Teliso-V has been associated with overlapping toxicities, including pulmonary embolism, colitis, hypertension, fatigue, and peripheral neuropathy, highlighting the need for careful sequencing and monitoring in combination regimens (43). Additionally, AI-based Clinical Decision Support Systems CDSS platforms may help mitigate these risks by integrating labs, imaging, and patient-reported data to trigger early safety alerts (82). However, the timing and order of drug administration are important; inadequate sequencing may lead to resistance or increased toxicity. As a solution, an in silico platform powered by DL and quantitative systems pharmacology (QSP) can simulate thousands of treatment combinations and dosing sequences using patient-level data from clinical trials and electronic health records. These simulations help in determining optimal regimens with minimal toxicity and maximum efficacy (83, 84). Furthermore, ML models using acute phosphoproteomic responses predicted NSCLC drug sensitivity better than the traditional biomarkers, while predicted drug combinations matched experimental Bliss synergy scores, supporting the role of ML-guided proteomics in optimizing therapies such as Teliso-V (85). Additionally, deep Q-learning techniques enable adaptive treatment sequencing, dynamically adjusting regimens in silico as resistance develops (86).

Effective use of Teliso-V in NSCLC demands adequate sequencing with biomarkers such as MET overexpression, as inappropriate administration in MET-negative tumors may result in no therapeutic benefit but increase toxicity risk, especially pulmonary adverse events (40). To support accurate prescribing, AI-enhanced CDSS combined with Electronic Medical Records (EMRs) can standardize Teliso-V use at the point of care. These systems automatically alert clinicians to order MET IHC or NGS testing when NSCLC is diagnosed, and flag eligibility for Teliso-V based on biomarker status and treatment history. Advanced CDSS modules can also screen for contraindications, such as prior lung conditions, and monitor for emerging adverse events by integrating real-time lab and clinical note data (87). In a broader oncology context, CDSS tools have been shown to increase adherence to biomarker-directed therapies and reduce prescription errors by over 30% (87). From a regulatory standpoint, several AI-based diagnostic tools have already been approved under the FDA’s Software as a Medical Device (SaMD) framework, especially in radiology and pathology (82). However, AI platforms designed for Teliso-V eligibility or sequencing are still under investigation as regulatory agencies like the FDA and EMA stress the importance of transparency, external validation, and clinician oversight before these tools can be used clinically (88). Therefore, AI-based applications in oncology, including potential (CDSS) for Teliso-V, should be seen as supportive tools rather than independent decision-makers (89). As AI models evolve, these systems will increasingly personalize dosing, sequence therapies, and suggest trial options based on dynamic patient profiles. Thus, EMR-integrated CDSS not only improve the safety and efficacy of Teliso-V administration but also promote evidence-based treatment delivery in NSCLC care.

Standard randomized controlled trials (RCTs) are often unable to address real-time developments, i.e., developing resistance or shifting biomarker profiles, a key limitation when assessing targeted therapies like Teliso-V in NSCLC (90). In this context, AI-powered adaptive trial designs promise a solution by allowing dynamic trial adjustments based on early response data, toxicity predictions, and patient classification. For example, ML algorithms can evaluate interim ctDNA dynamics or imaging features to identify non-responders early and reassign them to more effective treatments, reducing exposure to ineffective therapies (91). Moreover, AI can also predict adverse events using electronic health record (EHR) data and adjust dosage beforehand, improving safety without delay (92). Additionally, site selection algorithms that assess demographic, genomic, and referral data can boost the recruitment of patients by identifying regions having a greater number of MET-positive NSCLC cases, lowering trial delays and improving enrollment diversity (93).

Despite all the above factors, many clinical as well as technical challenges limit the implementation on a large scale. Clinically, many oncologists and support staff are not trained enough to understand AI-generated insights such as biomarker-driven treatment recommendations or toxicity forecasts (94). Additionally, skepticism persists due to concerns over data privacy, legal liability, and trust in algorithmic decision-making, especially when these decisions impact the treatment outcomes of patients (95). Technically, hospital EMRs, imaging systems, and AI platforms usually lack interoperability, making integration difficult (95). Moreover, data silos, fragmented, incomplete, or non-standardized datasets, further disturb the accuracy and reproducibility of algorithms, especially when real-time inputs like ctDNA levels or radiomic metrics are needed (96). Moreover, as mentioned above, regulatory and ethical frameworks have not fully caught up with the pace of AI, limiting real-time use in clinical decision-making due to concerns over accountability and patient consent (97). Overcoming these challenges demands combined efforts across multiple sectors, i.e., oncologists, data scientists, informatics experts, and pharmaceutical developers need to work together to create transparent, interoperable, and clinically validated AI tools according to regulatory standards while maintaining clinician oversight.

## Summary of trials and multidisciplinary potential

With the recent approval of Teliso-V by the FDA for NSCLC, this ADC has shown encouraging signs, being significantly more effective in MET-high populations. 172 adults diagnosed with non-squamous *EGFR*-wildtype NSCLC were given Teliso-V in stages I and II. The median duration of response was 8.3 months while the median overall survival was 14.5 months. Similarly, the median progression-free survival was 5.7 months. The effectiveness is highlighted by the fact that it shows a much higher ORR of 34.6% in MET-high and 22.9% in MET-intermediate populations based on the phase II LUMINOSITY trial, compared to other second line therapy drugs (40, 98, 99). Although patients experienced treatment-related adverse effects (TRAEs) like peripheral sensory neuropathy, peripheral edema and pneumonitis being the most common, the trial indicated that these side effects were manageable and rarely led to any serious complication (100). Furthermore, with the recent development of technology and AI being integrated in the field of medicine, AI has shown promising results in early diagnosis of not only the type but also the stage of cancer (26, 101). With algorithms like ML and DL being able to analyze multimodal data at incredibly high rates, AI can process radiomics and not only help to evaluate the efficacy of an immunotherapic drug, but also allow doctors to make a consistent and accurate decision for the best treatment plan (102, 103).

Showing a durable response and tolerable side effects, Teliso-V became the first approved therapy specifically targeting c-Met-overexpressing NSCLC. Similarly, flexible dosages and time interval of the drug not only help adjust the optimization, tolerability and individual’s metabolic needs, but could also be used potentially in a broader population (104). The effectiveness can be shown by the fact that phase III LUMINOSITY trial is being conducted which in the future could help us evaluate further the response as well as the safety and tolerability profile factor to treat EGFR- non-squamous NSCLC. Teliso-V, showing ADC’s mechanism has shown multidisciplinary potential in fields other than oncology such as Pathology and Molecular Diagnostics where immunohistochemistry SP44 assay was used in c-MET population categorization (40). Similarly, pneumonitis being presented as any-grade TRAE could involve pulmonology to tackle its toxicities. Being able to observe tumors and the treatment response via RECIST v1.1 as well as adjusting the dosages and time intervals of the drug highlights the importance of Radiology and Pharmacology respectively (40).

## Limitations

However, a few research gaps were seen in the results, one of them being non-randomization and the need for prospective randomized trial. The trial was unable to compare Teliso-V with standard treatments, resulting in doctors facing a dilemma whether the efficacy was validated. Furthermore, absence of a control arm, testing predominantly white population and the drug being tested on previously treated EGFR- non-squamous NSCLC has brought selection bias, some of which are said to be addressed in the Phase III LUMINOSITY trial (40). Additionally, the use of IHC-assay to measure c-MET overexpression has led to another limitation as it could only be stratified to just high and intermediate c-MET population, which in turn shows a conundrum while evaluating the ORR and OS. Improved and composite biomarkers in the future could not only help to evaluate the functional activity of c-MET protein accurately but also use alternative indicators such as MET gene alterations and EGFR mutations to precisely determine the type of NSCLC (105). Short follow-up duration and small group analysis could make doctors unable to evaluate the long-term side effects and generalize it to a broader population.

## Conclusion and future perspectives

In conclusion, AI integrated with ADCs like Teliso-V can show a promising efficacy in the coming years not only in terms of time management, but also the reliability and accuracy. The next decade holds the potential for ADCs such as Teliso-V to address the shortcomings of conventional treatments and enhance outcomes in treatment-resistant cancers. Furthermore, AI incorporated in oncology could not only help in the development of personalized medicines in the future, but could also optimize the affinity and efficacy of ADCs for better treatment (106). AI would not just be limited to screening cancers, but also be used to expand the development of “Intelligent ADCs” which could be used in real-time sensing and dynamic response (107).

## Glossary

NSCLC: Non-small Cell Lung Cancer
LUAC: Lung Adenocarcinoma
TKIs: Tyrosine Kinase Inhibitors
EGFR: Epidermal Growth Factor Receptor
KRAS: Kirsten rat sarcoma viral oncogene homolog
c-MET: Mesenchymal-epithelial Transcription Factor
AI: Artificial Intelligence
Teliso-V: Telisotuzumab Vedotin
ADC: Antibody-drug Conjugate
MMAE: Monomethyl Auristatin E
vcMMAE: Valine-citrulline MMAE
HGF: Hepatocyte Growth Factor
ML: Machine Learning
DL: Deep Learning
GRB2: Growth Factor Receptor-bound Protein 2
PI3K: Phosphoinositide 3-kinase
STAT3: Signal Transducer and Activator of Transcription 3
VEGF-C: Vascular Endothelial Growth Factor C
ELISA: Enzyme-linked Immunosorbent Essay
FACS: Fluorescence-activated Cell Sorting
PK: Pharmacokinetics
NLP: Natural Language Processing
CT: Computed Tomography
PET-CT: Positron-emission Tomography-CT
ICIs: Immune Checkpoint Inhibitors
PD-1: Programmed Cell Death Protein 1
PD-L1: Programmed Cell Death Ligand 1
IHC: Immunohistochemistry
TMB: Tumor Mutational Burden
TME: Tumor Microenvironment
FDA: Food and Drug Administration
q2w: Once every 2 weeks
ORR: Objective Response Rate
PFS: Progression-free Survival
OS: Overall Survival
DOR: Duration of Response
q3w: Once every 3 weeks
RP2D: Recommended Phase 2 Dose
OD: Once Daily
DCR: Disease-control Rates
AEs: Adverse Events
AUC: Area Under Curve
TILs: Tumor-infiltrating Lymphocytes
ctDNA: Circulating Tumor DNA
bTMB: Blood-based TMB
TMBRB: TMB Radiomic Biomarkers
ECOG: Eastern Cooperative Oncology Group
Rad: Radiomics
H-score: Histochemical Scoring
PCR: Polymerase Chain Reaction
RNA: Ribonucleic Acid
H&E: Hematoxylin and Eosin Stain
RECIST: Response Evaluation Criteria in Solid Tumors
NGS: Next-generation sequencing
QSP: Quantitative Systems Pharmacology
CDSS: Clinical Decision Support Systems
EMRs: Electronic Medical Records
RCTs: Randomized Control Trials
EHR: Electronic Health Records
TCGA: The Cancer Genome Atlas Program
CNNs: Convolutional Neural Networks
RF: Random Forest
XGB: Gradient Tree Boosting
SaMD: Software as a Medical Device
TRAEs: Treatment-related Adverse Events
CD31: Cluster of Differentiation 31
EMT: Epithelial-Mesenchymal Transition
ERK: Extracellular signal-regulated Kinase;OP-18, Oncoprotein 18.
